# Effect of a Static Magnetic Fields and Fluoride Ions on the Antioxidant Defense System of Mice Fibroblasts

**DOI:** 10.3390/ijms140715017

**Published:** 2013-07-18

**Authors:** Ewa Kurzeja, Agnieszka Synowiec-Wojtarowicz, Małgorzata Stec, Marek Glinka, Stanisław Gawron, Katarzyna Pawłowska-Góral

**Affiliations:** 1 Department of Food and Nutrition, Medical University of Silesia in Katowice, 8 Jednosci Street, 41-200 Sosnowiec, Poland; E-Mails: ekurzeja@sum.edu.pl (E.K.); mstec@sum.edu.pl (M.S.); kgoral@sum.edu.pl (K.P.-G.); 2 Research and Development Centre of Electrical Machines, 188 Rozdzienskiego Street, 40-203 Katowice, Poland; E-Mails: mag@iq.pl (M.G.); s.gawron@komel.katowice.pl (S.G.)

**Keywords:** static magnetic fields (SMF), fluoride, rat fibroblasts, redox homeostasis

## Abstract

The results of studies on the biological influence of magnetic fields are controversial and do not provide clear answers regarding their impact on cell functioning. Fluoride compounds are substances that influence free radical processes, which occur when the reactive forms of oxygen are present. It is not known whether static magnetic fields (SMF) cause any changes in fluoride assimilation or activity. Therefore, the aim of this work was to determine the potential relationship between magnetic field exposure to, and the antioxidant system of, fibroblasts cultured with fluoride ions. Three chambers with static magnetic fields of different intensities (0.4, 0.6, and 0.7 T) were used in this work. Fluoride ions were added at a concentration of 0.12 mM, which did not cause the precipitation of calcium or magnesium. The results of this study show that static magnetic fields reduce the oxidative stress caused by fluoride ions and normalize the activities of antioxidant enzymes, including superoxide dismutase (SOD), glutathione peroxidase (GPx), and catalase (CAT). Static magnetic fields modify the energy state of fibroblasts, causing an increase in the ATP concentration and a decrease in the MDA concentration. These results suggest that exposure to fluoride and an SMF improves the tolerance of cells to the oxidative stress induced by fluoride ions.

## 1. Introduction

As civilization becomes more advanced, people are increasingly exposed to artificial magnetic fields (MFs). For example, home appliances and medical devices are sources of MFs. This increase may lead to functional disturbances in various biological systems. The results of previous studies conducted on this topic, both *in vivo* and *in vitro*, are controversial and do not provide a clear answer regarding the impact of magnetic fields on cell functioning [1–3]. This lack of clarity is due to the use of magnetic fields of different intensities and different cell types.

Some research has shown that static magnetic fields (SMFs) do not influence cell culture growth, cell proliferation kinetics, or the cell cycle [4,5]. Other research has drawn attention to changes in the morphological structure of cells, such as their shape, surface modifications, such as blebs and microvilli, and structural changes in the membrane [6,7]. Štolfy *et al.* [8] demonstrated that human chondrocytes (HCH) subjected to an SMF with a flux density of 0.6 T show an increase in viability. Previous reports showing that the main target of magnetic field activity is the Ca^2+^ signaling pathway [9] are particularly important. Calcium plays a key role as a secondary information transmitter in cells. Its concentration controls life processes as different as cyclic nucleotide levels, hormone and neurotransmitter secretion, growth, differentiation, and death. Both induced and spontaneous apoptosis have been reported to be influenced by SMF exposure. However, some reports have failed to detect any apoptotic effect for SMFs [10,11].

Fluoride compounds are often investigated in relation to their impact on live organisms. Fluoride is an essential element, as proper functioning of bone tissue and teeth depends on its presence. If the quantity of fluoride supplied to the organism is greater than organism’s capacity for its excretion, the result is dental and skeletal fluorosis. In most cases, advanced forms of fluorosis occur in regions where the natural concentration of fluoride in the drinking water is very high [12,13]. However, bearing in mind the significant abundance of fluoride compounds in the natural environment, its presence in food, and its use in dentistry and medicine, chronic poisoning with fluoride must be taken into account. The latest reports indicate that fluoride toxicity is not limited to its impact on bones and teeth and affects all organs [14]. Many reports have shown that fluorides cause oxidative stress, lipid peroxidation, and changes in enzymatic antioxidant activities *in vivo* and *in vitro* [15].

Cells can react to static magnetic fields in many ways, including the production of free radicals. The aim of the present study was to determine the potential relationship between magnetic field exposure and the antioxidant system of fibroblasts cultured with fluoride ions.

## 2. Results and Discussion

Empirical data on the impact of static magnetic fields on live organisms, as opposed to electromagnetic fields, is limited and is generally restricted to fields with flux densities exceeding 1 T (T- tesla). SMFs are classified as weak (<1 mT), moderate (1 mT–1 T), strong (1–5 T), or ultra strong (>5 T) [16]. In this study, we used permanent magnets with moderate flux densities.

### 2.1. Effects of Fluoride on Mice Fibroblast Cultures

The participation of F^−^ in free radical processes, including those involving ROS, has been confirmed in numerous reports [17–19]. However, previous results concerning the activities of the antioxidant enzymes (superoxide dismutase SOD, glutathione peroxidase GPx, catalase CAT), and glutathione S-transferase (GST), the concentration of malondialdehyde (MDA), and the total antioxidant potential (ABTS) do not present a uniform picture. The observed differences are due to the cells type, of fluoride ion concentration, and testing time. A significant problem that is encountered when studying on the effect of fluoride ions *in vitro* and, presumably, also *in vivo*, is the fact that these ions bind to Mg^2+^ and Ca^2+^ ions, creating fluorides that are difficult to dissolve. Due to the low solvability of CaF_2_ and MgF_2_, when the concentration of F^−^ ions in the culture medium is high, these fluorides precipitate. This causes a decrease in the Mg^2+^ and Ca^2+^ concentration: the concentration of calcium fluoride, which is more difficult to dissolve, is the first to fall. In this situation, the observable effects result from the decrease in the cellular concentrations of the calcium and magnesium ions, not from the fluoride ion activity. In this study, the applied fluoride ion concentrations did not result in cell death rates that were higher than those in the control cultures and did not violate the integrity of the fibroblast cell membranes. This was also confirmed by the low LDH activity observed in the media mediums of the control and test cultures (results not shown).

**Figure 1:**
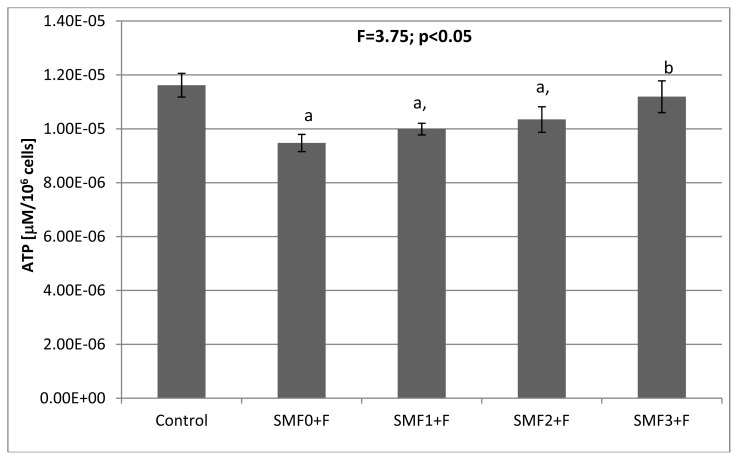
Effects of the static magnetic fields (SMF) and fluoride on adenosine triphosphate (ATP) concentration in fibroblasts. Results are presented as mean ± S.D. (*n* = 5); ^a^*p* < 0.05 *vs.* Control; ^b^*p* < 0.05 *vs.* SMF0 + F. Experimental conditions as per that described in Table 1.

**Figure 2:**
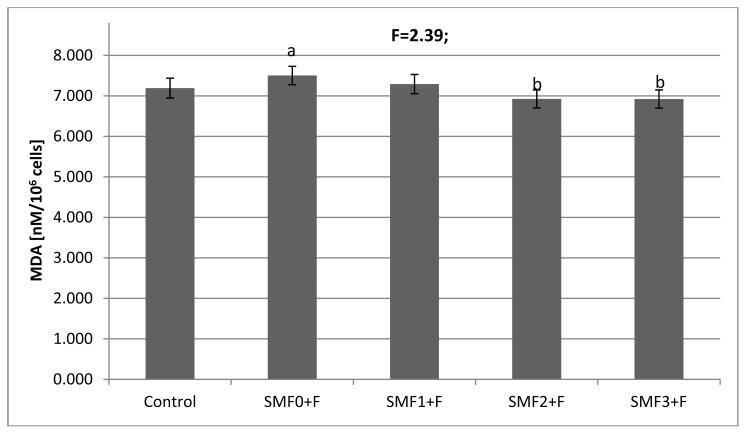
Effects of the static magnetic fields (SMF) and fluoride on malondialdehyde (MDA) concentration in fibroblasts. Results are presented as mean ± S.D. (*n* = 5); ^a^*p* < 0.05 *vs.* Control; ^b^*p* < 0.05 *vs.* SMF0 + F. Experimental conditions as per that described in Table 1.

**Figure 3:**
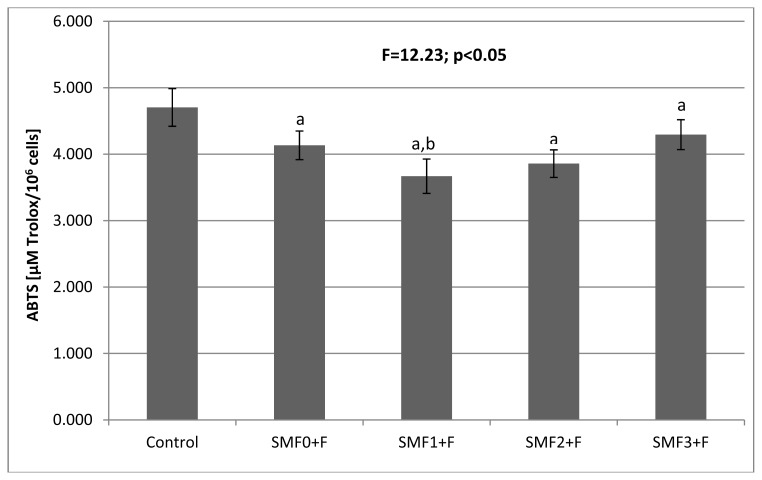
Effects of the static magnetic fields (SMF) and fluoride on antioxidant status (ABTS) in fibroblasts. Results are presented as mean ± S.D. (*n* = 5); ^a^*p* < 0.05 *vs.* Control; ^b^*p* < 0.05 *vs.* SMF0 + F. Experimental conditions as per that described in Table 1.

The F^−^ ions added to the medium (SMF0 + F, F^−^ = 0.12 mM) showed statistically a significant effect (*p* < 0.05) on the activities of the antioxidant enzymes: SOD and GPx, which were decreased by 28% and 20%, respectively, compared to the control culture (Table 1). The CAT and GST activities did not differ (*p* > 0.05) from the respective values obtained for the control culture. The changes in activities of SOD and GPx were accompanied by an 18% decrease in the ATP concentration (Figure 1), a ~9% increase in the MDA concentration (Figure 2), and a 12% reduction in the total antioxidant potential (Figure 3). These results clearly demonstrate that fluoride ions cause oxidative stress in fibroblasts.

### 2.2. Effects of Static Magnetic Fields and Fluoride on Mice Fibroblast Cultures

Our research on fibroblast cultures subjected to both fluoride and a static magnetic field showed statistically significant changes in the studied parameters compared to both the control and SMF0 + F groups (Table 1).

The activities of SOD, GPx, and CAT determined for the fibroblasts cultured with fluoride ions were increased in the presence of a static magnetic field compared to the SMF0 + F fibroblast cultures. This finding suggests that constant magnetic fields promote the normalization of antioxidant enzyme activities and reduce fluoride ion-induced oxidative stress.

Superoxide dismutase, which protects cells from the very reactive superoxide anion, plays a critical role in the ROS defense system. Around the positively charged SOD active site, there are charged amino acid residues, creating an electric field gradient that directs O^2 •−^ into site of the active enzyme. This mechanism has been termed ‘electrostatic guiding’ [20]. The observed decrease in SOD activity likely resulted from the attraction of fluoride instead of O^2 •−^ into the SOD active site, which may indicate competitive inhibition for SOD. Regardless of strength of the magnetic field used, a statistically significant difference (*p* < 0.05) was observed in the SOD activity. An increase in the magnetic field flux density was accompanied by an increase in the SOD activity. Comparison of the SOD activities for the SMF0 + F and SMF3 + F groups showed a statistically significant increase (*p* < 0.05) of ≈12% for the SMF3 + F group. The increased SOD activity that was observed in the cultures subjected to a SMF may indicate the inhibition of F^−^ flow to the SOD active site.

GPx and CAT are responsible for the decomposition of hydrogen peroxide. The activity of GPx was lower for SMF1 + F, and higher for SMF3 + F than that of the control culture by ≈14% and 11%, respectively. Comparison of GPx activity in relation to SMF0 + F shows its substantial increase, which corresponds with an increase in the magnetic field intensity. The CAT activities were not significantly different in the test groups compared to the control and SMF0 + F groups. Only in case of SMF3 + F and tests run in the chamber with a flux density equal to 0.7 T, a significant (*p* < 0.05) increase in the CAT activity compared to SMF0 + F was observed; this increase was ≈23%. The diverse changes in the GPx and CAT activities are because that GPx has greater affinity for hydrogen peroxide and CAT operates higher substrate concentrations.

The GST activities for all of the test cultures were higher than that of the control group. Comparison of GST activity in relation to SMF0 + F shows its substantial increase, which corresponds to an increase in the magnetic field intensity. This increased activity is most likely due to the ability of GST to inactivate the endogenous products of oxidative stress, in particular peroxides and aldehydes produced during lipid peroxidation.

There are few reports on the effect of SMFs on antioxidative defense parameters. In previous studies performed by Glinka *et al.* [5] and Kula *et al.* [21], human and mouse fibroblasts did not exhibit increased SOD, GPx, or CAT activity when magnetic fields with flux densities of 0.49 T and 128 mT were applied. On the other hand, studies on the effects of a 128 mT SMF on rats provide discrepant results. Ghodbane *et al.* [22] showed the decreased activity of GPx in the muscles and kidneys, and the increased activity of SOD in the liver with this SMF, while Amara *et al.* [23] noted the decreased activities of CAT, CuZn-SOD, and GPx in the livers and kidneys of the tested rats.

The presence of a static magnetic field also modified the energy state of the fibroblasts. Exposure to fluoride and a constant magnetic field caused changes in the cellular ATP concentration (Figure 1). In the SMF1 + F and SMF2 + F cultures, the ATP concentration was significantly (*p* < 0.05) lower than in that in the control group, whereas the ATP concentration in the SMF3 + F culture did not differ from that in the control group. Comparison of the results obtained for SMF0 + F indicates a substantial increase in the ATP concentration in cultures subjected to a static magnetic field. An increase in the ATP concentration in *Escherichia coli* and *Pseudomonas putida* bacteria subjected to a 17 mT SMF was previously reported by Filipič *et al.* [24].

Evaluation of lipid peroxidation in our fibroblast cultures showed that SMF exposure positively affected the MDA concentration, causing its decrease compared to the cultures subjected to fluoride ion-induced oxidizing stress. In the SMF2 + F and SMF3 + F cultures, the MDA concentration was ≈8% lower than that determined for SMF0 + F (Figure 2). Our data point to the existence an adaptive mechanism that is associated with a decrease in MDA, following exposure to a SMF. In contrast, Kabuto *et al.* [25] showed that exposure to a SMF of 1.5–300 mT has no effect on the accumulation of MDA in brain homogenates. In addition, HL-60 cells and lymphocytes exposed to a 7 mT SMF did not exhibit increased lipid peroxidation or increased MDA levels [26,27].

The antioxidant potential of cells cultured in a static magnetic field was substantially decreased in comparison to that of control culture (Figure 3). This change was inversely proportional to the flux density value. A greater magnetic field intensity was accompanied by a greater decrease in the total antioxidant potential. We did not note any significant changes in the antioxidant potential in the SMF2 + F and SMF3 + F cultures in comparison to the SMF0 + F culture. However, for the SMF1 + F culture, the antioxidant potential was lower than the SMF0 + F culture by ≈11%. These results proved the interference of fluoride ions in the antioxidant system of the tested fibroblasts. The reduction in the antioxidant potential was due to a decrease in the concentration of the hydrophilic antioxidants that are involved in free radical redox reactions. The presence of a static magnetic field did not have any effect on the antioxidant potential of the fibroblasts cultured with fluoride ions.

## 3. Experimental Section

### 3.1. Chemicals

All reagents were purchased from Sigma-Aldrich (St. Louis, MO, USA).

### 3.2. Magnetic Test Chamber Features

The magnetic chambers used to culture cells in a static magnetic field consisted of a ferromagnetic yoke, which constituted the bottom and cover of the chambers and permanent magnets. The chambers were enclosed by lateral, front, and back walls; the front wall was fitted with a window. The window dimensions corresponded to the lateral dimensions of a culture flask. Non-magnetic distance plates determined the inner dimensions of the chambers, which were matched to the culture flask dimensions. If the permanent magnets used were thin, then the distance plates were placed between the magnets and the ferromagnetic yoke. Their thickness corresponded to the magnet thickness.

The design of these test chambers allowed for uniform distribution of magnetic flux density over the measurement space of the flask. The magnetic field intensity was directly proportional to the strength of the magnetic field used, and neodymium magnets were used to generate the magnetic field. The materials used for the chambers are as follows: N42SH magnets, *B_r_* = 1.28–1.34 T, *H*_cB_ ≥ 955 kA/m, *H*_cJ_ ≥ 1512 kA/m, (BH)_max_ = 310–342 kJ/m^3^, S235JR steel and diamagnetic material. The chambers had a maximum operating temperature of 150 °C. Three chambers were used, with three different magnet sizes (6 mm, 11 mm, and 20 mm thick, respectively). The flux densities in the chambers were 0.4 (SMF1), 0.6 (SMF2), and 0.7 T (SMF3), respectively. The control culture chamber was not equipped with permanent magnets (steel was used instead), and a gauss meter showed an overall flux density of 0 T (SMF0) for this chamber.

### 3.3. Fibroblast Isolation

Fibroblasts were isolated from the tail and belly skin of a 60-day-old mouse, which was obtained from the Experimental Medicine Centre of Silesian Medical University. This study was approved by the Local Animal Experimentation Ethics Committee.

### 3.4. Cell Culture Conditions

Fibroblasts were cultured in 10 mL medium containing NaF and exposed to a static magnetic field (flux density of 0.4, 0.6, or 0.7 T) in the test chambers. The medium used was D MEM supplemented with inactive fetal bovine serum; 1000 U penicillin, 10 mg streptomycin and 25 μg amphotericin B per 1 mL medium with, and without, fluoride ions that were not exposed to a magnetic field (SMF0 + F and SMF0-F, respectively) were also grown. The fluoride ions concentration in the test medium was equal to 0.12 mM. The flasks were placed in the test chambers with a constant flux density for 4 days. The test and control cultures were cultured at 37 °C and 5% CO_2_, in a *Heraeus* incubator.

### 3.5. Cell Viability Assay

To assess cell and viability, the fibroblasts were stained with 0.4% trypan blue and counted (Countess Automated Cell Counter-Invitrogen, Carlsbad, CA, USA) Cell viability was measured by analyzing the trypan blue uptake [28].

### 3.6. Lactate Dehydrogenase (LDH) Release Assay

The LDH activity in the medium was determined for control and test cultures based on the Legrand *et al.* method [29], using test from (Sigma-Aldrich, St. Louis, MO, USA). The reduction of NAD^+^ to NADH, which is catalyzed by lactate dehydrogenase, was exploited in this assay. The LDH activity is reported as the percentage of the control value.

### 3.7. Preparation of Cell Homogenate

At the termination of each treatment, the cells were washed twice with ice-cold phosphate-buffered saline (PBS). The fibroblasts were mechanically homogenized using an Ultra-Turrax homogenizer (IKA Labortechnik, Staufen, Germany), in a flask placed on ice. The homogenization time was experimentally established, by assessing the effectiveness of the homogenization under a microscope. The resultant homogenates were then used in subsequent analyses.

### 3.8. Biochemical Analysis

All the studied biochemical parameters were recalculated for 10^6^ cells.

#### 3.8.1. Superoxide Dismutase (SOD) Activity Assay

SOD activity was estimated according to the method of Beauchamp and Fridovich [30]. This method employs xanthine and xanthine oxidase to generate superoxide radicals, which react with 2-(4-iodophenyl)-3-(4-nitrophenol)-5-phenyltetrazolium chloride (INT) to form a red formazan dye. The superoxide dismutase activity is then measured by the degree of inhibition of this reaction. The absorbance at 505 nm was recorded for the calculation of SOD activity. One unit (U) of SOD causes a 50% inhibition of the rate of reduction of INT under the conditions of this assay.

#### 3.8.2. Glutathione Peroxidase (GPx) Activity Assay

The GPx activity was measured according to the method of Paglia and Valentine [31]. In this method, glutathione peroxidase catalyzes the oxidation of glutathione by cumene hydroperoxide. In the presence of glutathione reductase and NADPH the oxidized glutathione is immediately converted to its reduced form with the concomitant oxidation of NADPH to NADP^+^. The decrease in absorbance at 340 nm was measured.

#### 3.8.3. Catalase (CAT) Activity Assay

The CAT activity was measured using the Catalase Assay Kit (Cayman Chemical, Ann Arbor, MI, USA). The method is based on the reaction of CAT with methanol in the presence of an optimal concentration of H_2_O_2_. The formaldehyde produced is measured spectrophotometrically with 4-amino-3-hydrazino-5-mercapto-1,2,4-triazole (Purpald) as the chromogen. Purpald specifically forms a bicyclic heterocycle with aldehydes, which upon oxidation changes from colorless to purple.

#### 3.8.4. Transferase (GST) Activity Assay

The GST activity was measured using the Glutathione S-Transferase Assay Kit (Cayman Chemical, Ann Arbor, MI, USA). This test determines the total GST activity (cytosolic and microsomal) by measuring the conjugation of 1-chloro-2,4-dinitrobenzene (CDNB) with reduced glutathione. This conjugation is accompanied by an increase in the absorbance at 340 nm and the increase is directly proportional to the GST activity in the sample.

#### 3.8.5. Determination of the Total Antioxidant Status (ABTS)

The total antioxidant status was measured using the ABTS^•+^ radical cation. The technique for the generation of ABTS^•+^ involves the direct production of the blue/green ABTS^•+^ chromophore through a reaction between ABTS and potassium persulfate. The addition of antioxidants to the pre-formed radical cation reduces it to ABTS, to an extent and on a time-scale that depends on the antioxidant activity, the concentration of the antioxidant and the duration of the reaction. The extent of decolorization as percentage inhibition of the ABTS^•+^ radical cation is determined as a function of concentration and time and is calculated relative to the reactivity of Trolox. The decrease in the absorbance at 734 nm was measured.

#### 3.8.6. Lipid Peroxidation Assay

Lipid peroxidation (as malondialdehyde, MDA) level was measured using the Bioxytech MDA-586 assay (OxisResearch, Foster City, CA, USA). This method is based on the reaction of a chromogenic reagent, *N*-methyl-2-phenylindole (NPMI) with MDA at 45 °C. One molecule of MDA reacts with two molecules of NPMI to yield a stable carbocyanine dye with maximum absorption at 586 nm. In the MDA assay, a calibration curve is prepared using at MDA standard.

#### 3.8.7. Determination of Adenosine Triphosphate (ATP) Concentration

The measurement of the ATP concentration was performed using the ATPlite 1step test (PerkinElmer, Waltham, MA, USA). The ATPlite 1step assay system is based on the production of light caused by the reaction of ATP with luciferase and d-luciferin.

### 3.9. Statistical Analysis

All data are expressed as the mean ± standard deviation of five separate experiments. An ANOVA and Tukey’s *post hoc* test were used to evaluate the results of the experiments. The statistical calculations were performed using Statistica 10.0 (Version 10.0, StatSoft^®^, Cracow, Poland), and the statistical significance was defined at *p* < 0.05.

## 4. Conclusions

In this study, we demonstrated that a moderate SMF modifies the redox homeostasis of fibroblasts exposed to fluoride ions. Therefore, we conclude that in locations with a high risk of fluoride exposure through water and food SMFs do not enhance the toxic free radical activity of fluoride ions.

## Figures and Tables

**Table 1:** Effects of the static magnetic fields (SMF) and fluoride on antioxidative defense parameters in fibroblasts.

	SOD [U/10^6^ cells]	GPx [U/10^6^ cells]	CAT [nM/min/10^6^ cells]	GST [nM/min/10^6^ cells]
Control	0.78 ± 0.024	627.6 ± 2.07	10.36 ± 0.86	68.73 ± 3.07
SMF0 + F	0.56 ± 0.018 a	503.6 ± 24.05 a	8.66 ± 0.89	70.15 ± 2.67
SMF1 + F	0.56 ± 0.024 a	541.7 ± 2.77 a,b	8.72 ± 0.87	74.78 ± 1.06 a,b
SMF2 + F	0.58 ± 0.013 a	615.4 ± 26.12 b	10.06 ± 0.59	76.54 ± 1.69 a,b
SMF3 + F	0.63 ± 0.010 a,b	695.2 ± 4.28 a,b	10.69 ± 0.85 b	83.16 ± 1.12 a,b

Results are presented as mean ± S.D. (*n* = 5);

a *p* < 0.05 *vs.* Control;

b *p* < 0.05 *vs.* SMF0 + F; Control, control culture without magnet (flux density 0T) and without fluoride ions; SMF0 + F, control culture with fluoride ions (0.12 mM) and without magnet (flux density 0 T); SMF1 + F, culture with fluoride ions (0.12 mM) and with magnet (flux density 0.4 T); SMF2 + F, culture with fluoride ions (0.12 mM) and with magnet (flux density 0.6 T); SMF3 + F, culture with fluoride ions (0.12 mM) and with magnet (flux density 0.7 T).

## References

[1] Todorović D., Mirčić D., Ilijin L., Mrdaković M., Vlahović M., Prolić Z., Mataruga V.P. (2012). Effect of magnetic fields on antioxidative defense and fitness-related traits of *Baculum extradentatum* (Insecta, Phasmatodea). Bioelectromagnetics.

[2] Lee B.C., Johng H.M., Lim J.K., Jeong J.H., Baik K.Y., Nam T.J., Lee J.H., Kim J., Sohn U.D., Yoon G. (2004). Effect of extremely low frequency magnetic field on the antioxidant defense system in mouse brain: a chemiluminescence study. J. Photochem. Photobiol. B.

[3] Sahebjamei H., Abdolmaleki P., Ghanati F. (2007). Effects of magnetic field on the antioxidant enzyme activities of suspension-cultured tobacco cells. Bioelectromagnetics.

[4] Miyakoshi J. (2005). Effects of a static magnetic field at the cellular level. Prog. Biophys. Mol. Biol.

[5] Glinka M., Gawron S.A., Sieroń A., Pawłowska-Góral K., Cieślar G., Sieroń-Stoltny K. (2013). Action of the static magnetic fields on the antioxidant activity in the fibroblasts’ culture. Prz. Elektrotech.

[6] Chionna A., Dwikat M., Panzarini E., Tenuzzo B., Carlà E.C., Verri T., Pagliara P., Abbro L., Dini L. (2003). Cell shape and plasma membrane alterations after static magnetic fields exposure. Eur. J. Histochem.

[7] Chionna A., Tenuzzo B., Panzarini E., Dwikat M., Abbro L., Dini L. (2005). Time-dependent modifications of Hep G2 cells during exposure to Static Magnetic Fields. Bioelectrmagnetics.

[8] Štolfa S., Škorvánek M., Štolfa P., Rosocha J., Vaško G., Sabo J. (2007). Effects of static magnetic field and pulsed electromagnetic field on viability of human chondrocytes *in vitro*. Physiol. Res..

[9] Gartzke J., Lange K. (2002). Celluar target of weak magnetic fields: Ionic conduction along actin filaments of microvilli. Am. J. Physiol..

[10] Teodori L., Gohde W., Valente M.G., Tagliaferri F., Coletti D., Perniconi B., Bergamaschi A., Cerella C., Ghibelli L. (2002). Static magnetic fields affect calcium fluxes and inhibit stress-induced apoptosis in human glioblastoma cells. Cytometry.

[11] Teodori L., Grabarek J., Smolewski P., Ghibelli L., Bergamaschi A., de Nicola M., Darzynkiewicz Z. (2002). Exposure of cells to static magnetic fields accelerates loss of integrity of plasma membrane during apoptosis. Cytometry.

[12] Fawell J., Bailey K., Chilton J., Dahi E., Fewtrell L., Magara Y. (2006). Fluoride in Drinking-Water.

[13] Narayanaswamy M., Piler M.B. (2010). Effect of maternal exposure of fluoride on biometals and oxidative stress parameters in developing CNS of rat. Biol. Trace Elem. Res.

[14] Cicek E., Aydin G., Akdogan M., Okutan H. (2005). Effects of chronic ingestion of sodium fluoride on myocardium in a second generation of rats. Hum. Exp. Toxicol.

[15] Pawłowska-Góral K., Pilawa B. (2011). Detection of free radicals formed by *in vitro* metabolism of fluoride using EPR spectroscopy. Toxicol. In Vitro.

[16] Dini L., Abbro L. (2005). Bioeffects of moderate-intensity static magnetic fields on cell cultures. Micron.

[17] Agalakova N.I., Gusev G.P. (2012). Fluoride induces oxidative stress and ATP depletion in the rat erythrocytes *in vitro*. Environ. Toxicol. Pharmacol.

[18] Altıntaş L., Eşsiz D., Eraslan G., İnce S., Arslanbaş E. (2010). Prophylactic effect of *N*-acetylcysteine against sodium fluoride-induced blood oxidative stress in mice. Food Chem. Toxicol.

[19] Nabavi S.M., Nabavi S.F., Eslami S., Moghaddam A.H. (2012). *In vivo* protective effects of quercetin against sodium fluoride-induced oxidative stress in the hepatic tissue. Food Chem.

[20] Lévêque V.J., Vance C.K., Nick H.S., Silverman D.N. (2001). Redox properties of human manganese superoxide dismutase and active-site mutants. Biochemistry.

[21] Kula B., Dróżdż M. (1996). A study on magnetic field effects on fibroblast cultures. Part 2. The evaluation of the effects of static and extremely low frequency ELF magnetic fields on free-radical processes in fibroblast cultures. Bioelectrochem. Bioenerg.

[22] Ghodbane S., Amara S., Garrel C., Arnaud J., Ducros V., Favier A., Sakly M., Abdelmelek H. (2011). Selenium supplementation ameliorates static magnetic field-induced disorders in antioxidant status in rat tissues. Environ. Toxicol. Pharmacol.

[23] Amara S., Abdelmelek H., Garrel C., Guiraud P., Douki T., Ravanat J-L., Favier A., Sakly M., Rhouma B. (2007). Zinc supplementation ameliorates static magnetic field-induced oxidative stress in rat tissues. Environ. Toxicol. Pharmacol..

[24] Filipič J., Kraigher B., Tepuš B., Kokol V., Mandic-Mulec I. (2012). Effects of low-density static magnetic fields on the growth and activities of wastewater bacteria *Escherichiacoli* and *Pseudomonas putida*. Bioresour. Technol.

[25] Kabuto H., Yokoi I., Ogawa N., Mori A., Liburdy R.P. (2001). Effects of magnetic fields on the accumulation of thiobarbituric acid reactive substances induced by iron salt and H_2_O_2_ in mouse brain homogenates or phosphotidylcholine. Pathophysiology.

[26] Ishisaka R., Kanno T., Inai Y., Nakahara H., Akiyama J., Yoshioka T., Utsumi K. (2000). Effects of a magnetic fields on the various functions of subcellular organelles and cells. Pathophysiology.

[27] Jajte J., Grzegorczyk J., Zmyślony M., Rajkowska E. (2002). Effect of 7 mT static magnetic field and iron ions on rat lymphocytes: apoptosis, necrosis and free radical processes. Bioelectrochemistry.

[28] Philips D.J. (1978). Dye Exclusion Test for Cell Viability. Tissue, Culture, Methods and Application.

[29] Legrand C., Bour J.M., Jacob C., Capiaumont J., Martial A., Marc A., Wudtke M., Kretzmer G., Demangel C., Duval D. (1992). Lactate dehydrogenase (LDH) activity of the cultured eukaryotic cells as marker of the number of dead cells in the medium. J. Biotechnol.

[30] Beauchamp C., Fridovich I. (1971). Superoxide dismutase: Improved assays and an assay applicable to acrylamide gels. Anal. Biochem.

[31] Paglia D.E., Valentine W.N. (1967). Studies on the quantitative and qualitative characterization of erythrocyte glutathione peroxidase. J. Lab Clin. Med.

